# Supplementary figures and images for: p73 is required for vessel integrity controlling endothelial junctional dynamics through Angiomotin
Source: Cell Mol Life Sci. 2022 Oct 1;79(10):535. doi: 10.1007/s00018-022-04560-3 (PMC9525397; doi:10.1007/s00018-022-04560-3)

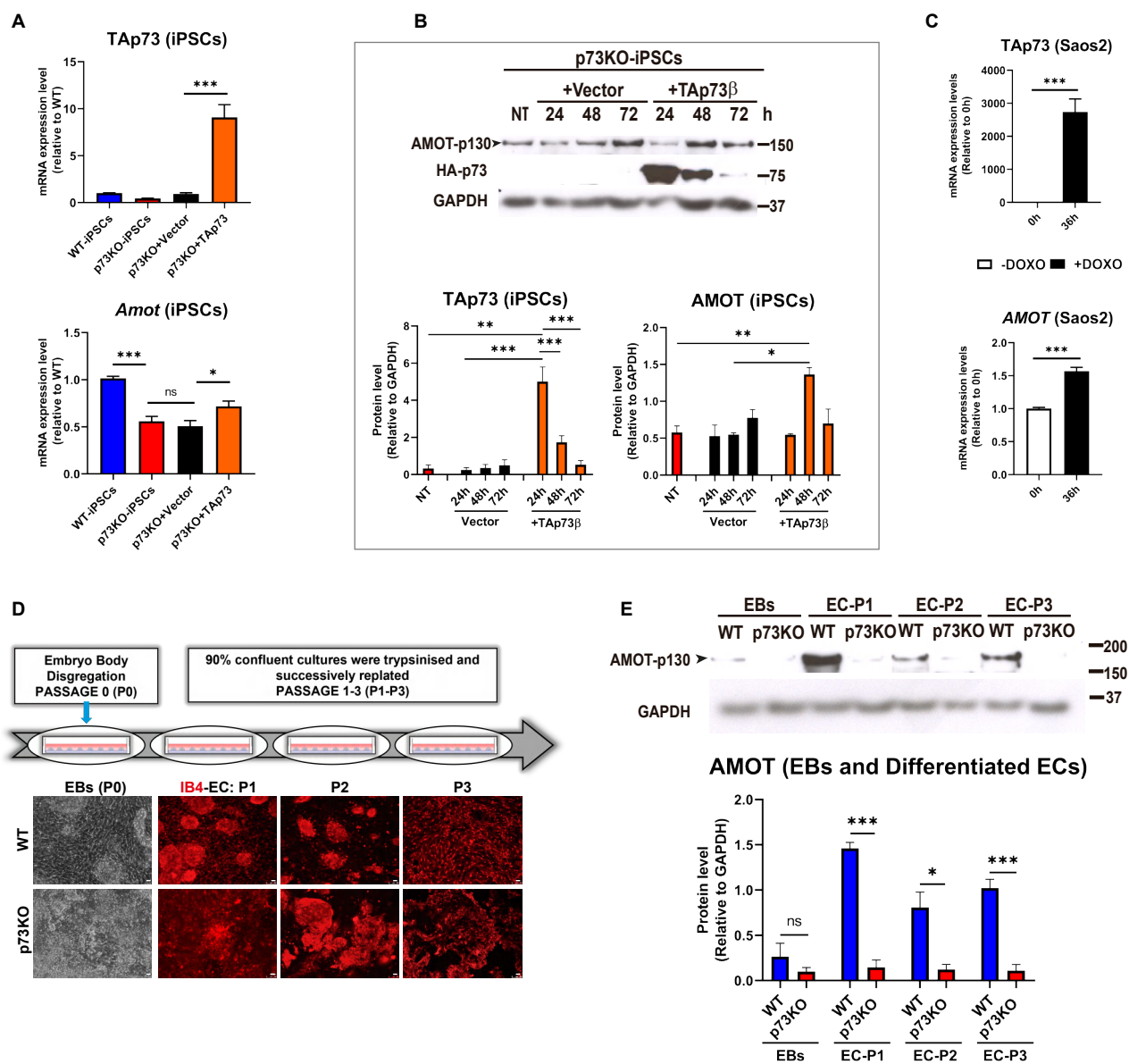

Supplement: Supplementary file 1 — Supplementary Fig. 1. p73 regulates AMOT levels in different cell types. A, B Correlation of Amot and TAp73 expression in p73KO-iPSCs. Amot RNA (A) and protein (B) levels increased following TAp73 ectopic expression. For transfection experiments, iPSCs were seeded (3.7 × 10^4^ cells/cm2) on Matrigel™ (Corning, #356231) coated plates and transfected with an empty Vector or a TAp73 expression plasmid using Lipofectamine™ 3000 Transfection Reagent (Invitrogen, # L300008) following the manufacturer’s instructions. Cells were analyzed 24 h after transfection (A) or up to 72 h after transfection (B). C TAp73 and AMOT expression analysis by qRT-PCR in HA-TAp73β-Saos2-Tet-On following Doxycycline treatment (–/ + DOXO), which induces TAp73 expression. Bars in (A, B, C) represent the mean ± SEM of three independent experiments. *p < 0.05, **p < 0.01, ***p < 0.001, ns: no significant. D Schematic drawing of the 2D-endothelial differentiation assay and representative phase-contrast and fluorescence microscopy images. EBs of the studied genotypes were disaggregated and plated (5 × 10^4^ cells/cm2) in EB medium (DMEM/GlutaMAX™-Gibco, #61965026, 25 mM HEPES, 1.2 mM sodium pyruvate, 19 mM monothioglycerol, 15% FBS) supplemented with 50 ng/ml VEGF-A (Peprotech, #450-32), on 0.1% gelatine-coated plates (non-endothelial, Passage 0). After 72-96 h hours in the presence of VEGF-A, 90% confluent cultures were trypsinized and replated on 0.1% gelatine-coated plates (endothelial differentiated cells, Passage 1). This step was repeated twice (Passage 2, 3). The cells were stained with biotinylated IB4 (2 μg/ml) to demonstrate their endothelial nature. Scale bar: 50 and 25 μm, respectively. E AMOT expression was analyzed by western blot in WT and p73KO undifferentiated cells (EBs) and quantified at various passages after endothelial differentiation (P1-P3) (PDF 316 KB) [file 18_2022_4560_MOESM1_ESM.pdf]

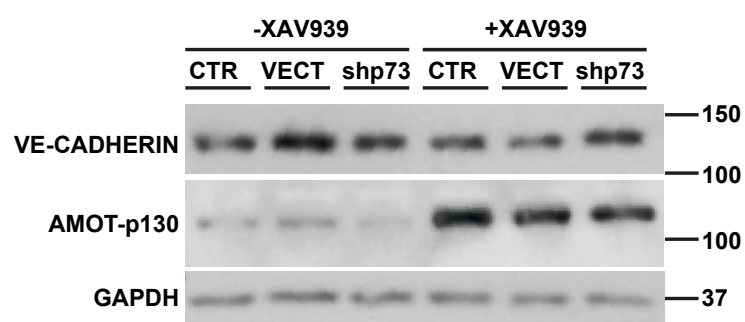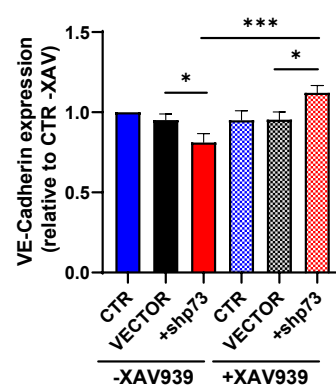

Supplement: Supplementary file 2 — Supplementary Fig. 2. p73 knockdown has only a slight effect on VE-cadherin expression. Western blot analysis of total VE-CADH in non-infected control HUVEC (CTR), and in cells infected with a non-silencing empty vector (VECT, VECTOR) or with an shRNA targeting p73 (+ shp73). Following p73 knockdown (48 h), cells were treated with XAV939 for 24 h. Treatment with the tankyrase inhibitor markedly induced AMOTp-130 expression. Only a small decrease in total VE-CADH expression was detected upon p73KD, although it was significantly recovered upon AMOT stabilization by XAV939 treatment. The graph shows the mean ± SEM of three independent experiments yielding similar results (PDF 261 KB) [file 18_2022_4560_MOESM2_ESM.pdf]

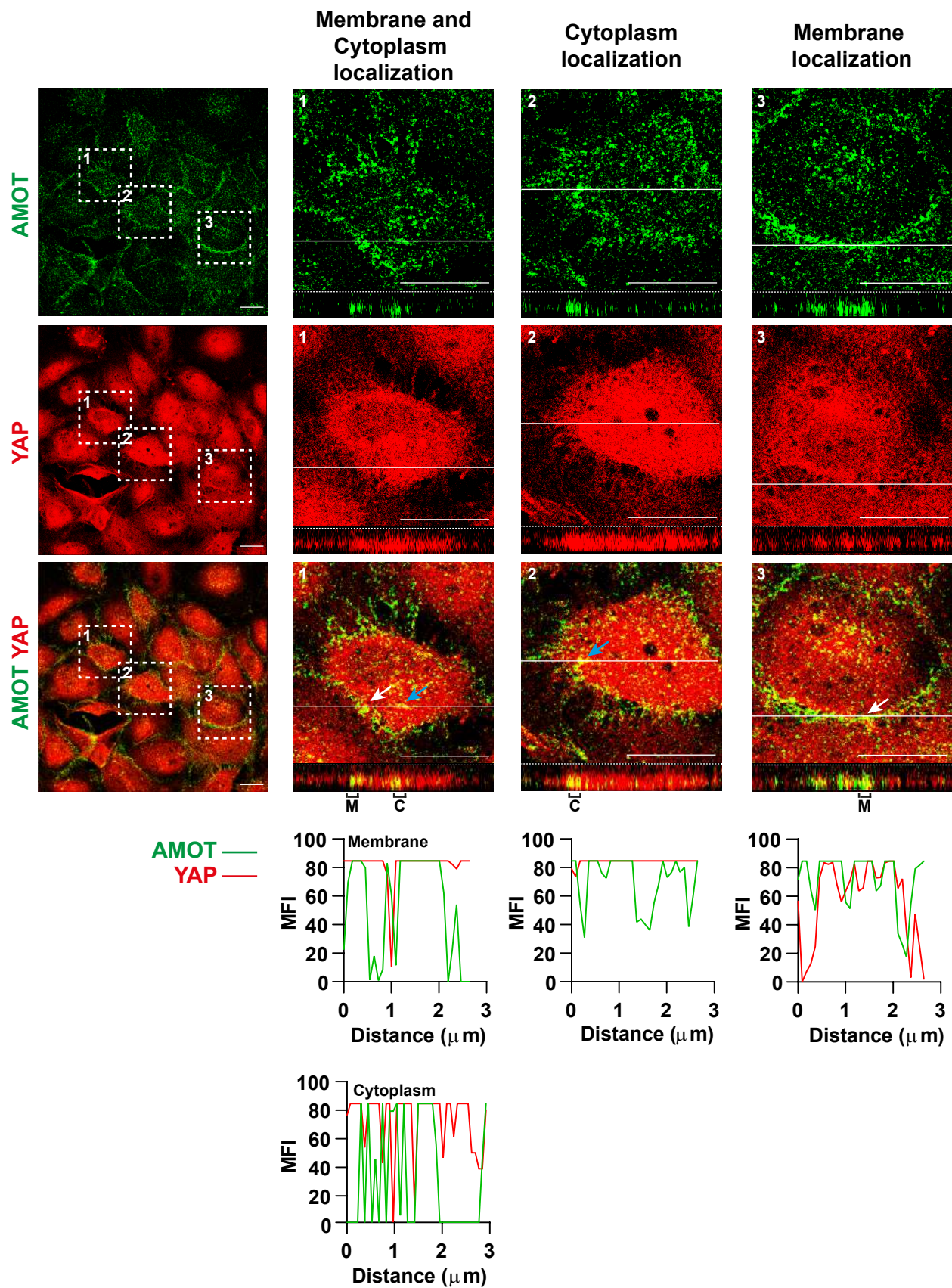

Supplement: Supplementary file 3 — Supplementary Fig. 3. AMOT colocalizes with YAP at the plasma membrane and in the cytoplasm. Detailed analysis of selected areas (white dashed squares 1, 2, 3) from Fig. 5 confocal micrographs showing confluent monolayers of p73KD cells treated with the tankyrase inhibitor XAV939. Cells were immunostained for AMOT (green) and YAP (red). Orthogonal projections of the magnified areas (1, 2, 3) show AMOT staining at the plasma membrane (M bracket) or the cytoplasm (C bracket) colocalizing with YAP. The histograms represent the mean fluorescence intensity (MFI) profiles of AMOT and YAP expression in the regions indicated by brackets at the orthogonal projections. Scale bar: 20 µm (PDF 1300 KB) [file 18_2022_4560_MOESM3_ESM.pdf]

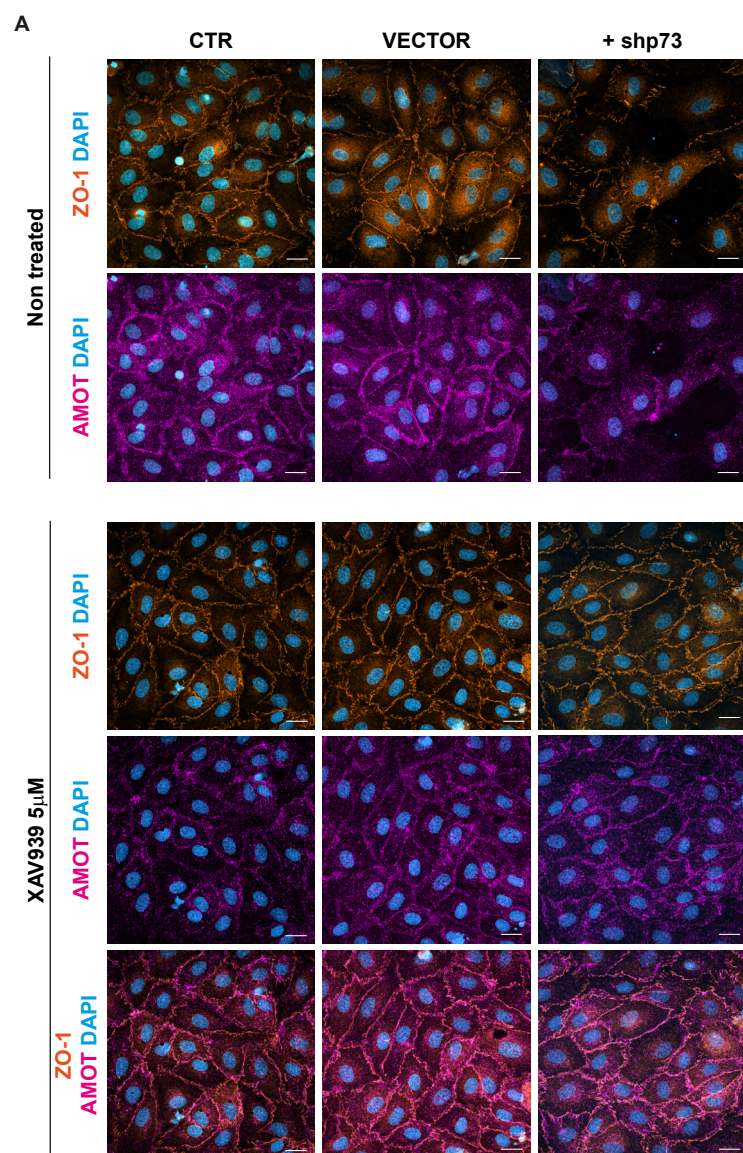

Supplement: Supplementary file 4 — Supplementary Fig. 4. AMOT is a p73-downstream effector for the regulation of tight junctions. Stabilization of endogenous AMOT levels by XAV939 treatment restores the tight junction defects induced by the p73KD. AMOT (pink) restoration rescues the morphology of TJs as shown by ZO-1 (orange) staining. Nuclei were counterstained with DAPI (blue). Scale bar: 20 µm (PDF 1034 KB) [file 18_2022_4560_MOESM4_ESM.pdf]
