# Supplementary material for: p73 is required for vessel integrity controlling endothelial junctional dynamics through Angiomotin
Source: Cell Mol Life Sci. 2022 Oct 1;79(10):535. doi: 10.1007/s00018-022-04560-3 (PMC9525397; doi:10.1007/s00018-022-04560-3)
Supplement: Supplementary file 5 — Supplementary Table 1. List of DEGs identified by RNA-seq in p73KO-iPSCs after TAp73β ectopic expression. (PDF 151 KB) [file 18_2022_4560_MOESM5_ESM.pdf]

Supplementary Table 1. List of DEGs identified by RNA-seq in p73KO-iPSCs after TAp73β ectopic expression. DEGs were consider significant at p-adj<0.05

Gene	log2FC	p-adj
Krt14	2,69	9,03E-96
Foxj1	2,55	1,45E-92
Perp	1,68	4,49E-71
Cdkn1c	1,42	1,66E-34
Cemip	1,59	3,63E-32
Itgb4	1,45	4,86E-32
Mdm2	0,91	1,91E-31
Dst	0,83	9,87E-22
Cbr2	1,09	1,27E-17
Junb	0,93	8,71E-17
Bcam	0,62	2,74E-16
Sord	1,01	1,24E-15
Ngfr	0,92	1,64E-14
Trp73	1,04	2,04E-14
Nptxr	1,05	5,21E-14
Gata6	0,96	2,44E-13
Idh1	0,69	3,38E-13
Pak6	1,00	1,10E-12
Wnt9a	0,94	3,01E-12
Vwa1	0,96	6,39E-12
Anxa8	0,97	7,04E-12
Jag2	0,91	1,03E-11
Ass1	0,49	1,98E-11
Cd109	0,92	2,78E-11
Itga6	0,54	3,59E-11
Wnt4	0,88	9,55E-11
Krt17	0,91	1,22E-10
Fermt1	0,93	1,31E-10
Cx3cl1	0,93	1,80E-10
Cotl1	0,76	2,57E-10
Slc38a3	0,87	4,19E-10
Amotl1	0,69	1,45E-09
Mfge8	0,58	2,15E-09
Scube3	0,81	2,15E-09
Col18a1	0,42	8,79E-09
Erf	0,48	8,88E-09
Lama5	0,54	1,18E-08
Cic	0,49	4,21E-08
Pard6g	0,81	6,22E-08
Lars2	0,36	8,10E-08
Apoe	0,43	8,14E-08
Gm26917	0,46	1,15E-07
Fermt3	0,70	1,95E-07
Tjp3	0,77	4,55E-07
Capn1	0,65	4,86E-07
Pou4f3	0,65	4,86E-07
Card10	0,69	6,49E-07
Cacna2d2	0,70	6,51E-07
Sytl1	0,74	6,51E-07
Col7a1	0,75	1,18E-06

Gene	log2FC	p-adj
Txnip	-0,40	2,12E-06
Fam101b	0,71	2,46E-06
Galnt18	0,67	2,88E-06
Coro6	0,59	4,19E-06
Tgfbr1	0,61	5,49E-06
Bmp7	0,68	5,71E-06
Zfp42	-0,35	6,14E-06
Plch2	0,63	9,10E-06
Bbx	0,56	9,13E-06
Fgf4	-0,41	9,13E-06
Fxyd3	0,51	9,96E-06
Nid2	0,39	9,98E-06
Nanog	-0,35	1,09E-05
Gas6	0,69	1,19E-05
Exoc3l4	0,62	1,23E-05
Fam129b	0,40	1,23E-05
Phc1	-0,32	1,31E-05
Rapgefl1	0,65	1,39E-05
Tnrc18	0,55	1,67E-05
Krtdap	0,60	2,00E-05
Krt19	0,67	2,82E-05
Pdgfa	0,57	2,82E-05
Tdgf1	-0,31	3,71E-05
Lamb1	0,46	4,00E-05
Ldlrap1	0,62	4,31E-05
Mcam	-0,48	4,31E-05
Mbp	0,64	4,51E-05
Ivl	0,46	5,15E-05
Nr0b1	-0,44	5,21E-05
Fam83g	0,65	6,19E-05
Tagln2	0,38	8,23E-05
Frmd4b	0,64	9,51E-05
Rdh10	0,64	9,51E-05
Spp1	-0,31	9,51E-05
Hr	0,63	1,07E-04
Ptpn13	0,57	1,07E-04
Wnt7b	0,54	1,17E-04
Esrrb	-0,29	1,20E-04
Spon2	0,49	1,36E-04
Tex261	0,49	1,36E-04
Aldh1a3	0,61	1,37E-04
Vgf	0,62	1,37E-04
mt-Atp8	0,44	1,39E-04
Eef2k	0,59	1,54E-04
Dnmt3a	-0,35	1,65E-04
Irgm1	-0,44	1,68E-04
Sdc4	0,38	1,97E-04
Mir6236	0,48	2,00E-04
Gm15662	0,42	2,06E-04
Zfp57	-0,33	2,06E-04

Gene	log2FC	padj
Rnf144b	0,68	1,18E-06
Kremen1	0,68	1,33E-06
Jarid2	-0,33	1,41E-06
Gprc5c	0,74	1,46E-06
Limk2	0,48	1,85E-06
Eps8l2	0,59	3,26E-04
Ece1	0,44	3,35E-04
Abhd17c	0,46	3,36E-04
Wnt3a	0,51	3,42E-04
Mkrn1	-0,26	3,46E-04
Adam8	0,59	3,56E-04
Fam83f	0,47	3,57E-04
Nbeal2	0,57	3,78E-04
Pkp3	0,58	3,78E-04
Dusp2	0,46	4,11E-04
Tmppe	0,52	4,29E-04
Il1rl2	0,45	4,33E-04
Coro7	0,58	4,50E-04
Sncg	0,53	4,91E-04
Pltp	0,55	4,97E-04
Lamb3	0,55	4,98E-04
RP23-473E2(	0,53	6,51E-04
Slc12a4	0,44	6,61E-04
Fat2	0,45	7,53E-04
Sertad4	0,52	9,01E-04
Glb1	0,48	9,43E-04
Pim1	-0,38	9,64E-04
Cpa4	0,37	1,07E-03
Pdia6	0,27	1,25E-03
Dmkn	0,54	1,28E-03
Vwa2	0,55	1,29E-03
Sept1	-0,33	1,43E-03
Bcl9l	0,54	1,47E-03
Oasl2	-0,55	1,63E-03
Ddx58	-0,36	1,71E-03
Hagh	0,54	1,71E-03
Isg15	-0,53	1,71E-03
Myrf	-0,43	1,71E-03
Hspg2	0,34	1,77E-03
Ifit1	-0,55	1,77E-03
Lima1	0,47	2,03E-03
Lamc2	0,45	2,06E-03
Rcan1	0,50	2,17E-03
Bend4	0,41	2,46E-03
Spry4	-0,38	2,52E-03
Mark4	0,48	2,68E-03
Chst3	0,47	2,74E-03
Krt5	0,37	2,78E-03
Ptp4a3	-0,45	2,78E-03
Cebpa	0,44	2,90E-03
Elmsan1	0,44	3,03E-03
Mvb12b	0,52	3,03E-03
Ubald2	0,34	3,08E-03
Mafb	0,40	3,13E-03
Parp12	-0,44	3,16E-03

Gene	log2FC	padj
Sema3f	0,57	2,10E-04
Pxn	0,47	2,90E-04
Sept9	0,37	3,00E-04
Dusp7	0,59	3,07E-04
Csrp1	0,38	3,23E-04
Efs	0,46	3,94E-03
Cmip	0,36	3,99E-03
Slc7a1	0,34	4,30E-03
Phyhip	0,42	4,38E-03
Dppa5a	-0,21	4,68E-03
L1td1	-0,21	4,68E-03
P2ry1	0,37	4,68E-03
Dab2ip	0,37	4,70E-03
Mt2	-0,25	4,80E-03
Myh9	0,27	4,80E-03
Ngfrap1	0,29	4,80E-03
Marcksl1	0,26	4,84E-03
Nxn	0,43	4,87E-03
Jup	0,29	5,55E-03
Glul	-0,30	5,68E-03
Plxnb2	0,33	5,77E-03
Efnb1	0,48	6,37E-03
Slc29a1	-0,29	6,52E-03
Itpkb	0,47	6,57E-03
Parp9	-0,46	6,59E-03
AI661453	0,46	7,20E-03
Gjb3	0,35	7,42E-03
Dag1	0,26	8,34E-03
Cyp26b1	0,40	8,48E-03
Rgs16	-0,48	8,48E-03
Fads2	0,43	8,72E-03
Pcsk5	0,32	9,00E-03
Mmp9	0,43	9,13E-03
Rbpms2	-0,31	9,18E-03
Atp13a3	0,27	9,20E-03
Sall1	-0,34	9,23E-03
Nav2	0,33	9,36E-03
Slc7a3	-0,32	9,42E-03
Trim47	0,45	9,58E-03
Il6ra	0,45	9,59E-03
Hyou1	0,26	9,81E-03
Irf6	0,48	9,81E-03
Hes2	0,34	9,95E-03
Stard8	0,49	9,95E-03
Pou5f1	-0,22	9,98E-03
Grem1	-0,38	0,010
Ppp1r13l	0,48	0,010
Cd151	0,36	0,010
Pvrl1	0,46	0,010
Wnt11	0,35	0,011
Nasp	-0,20	0,011
Phldb1	0,45	0,012
Sfn	0,42	0,012
Hyal2	0,42	0,012
Ttc7b	0,42	0,012

Gene	log2FC	padj
Rarg	-0,30	3,25E-03
Cd276	0,47	3,56E-03
Ccnd1	-0,34	3,66E-03
Fam60a	-0,27	3,74E-03
Rnase4	0,45	3,74E-03
Nsmaf	0,43	0,014
Tmem94	0,44	0,014
Fam83h	0,42	0,014
Hip1r	0,47	0,014
Rgs3	0,44	0,015
Alox12	0,35	0,016
Dsp	0,39	0,016
Hmgxb4	-0,27	0,016
Oas2	-0,46	0,018
Itga3	0,46	0,019
Bmp6	0,36	0,019
Flnb	0,28	0,019
Enah	-0,21	0,019
Etv5	-0,23	0,020
2900026A02F	0,46	0,020
Dhrs3	0,37	0,020
Src	0,36	0,020
Dhx16	-0,25	0,021
Slc25a4	0,29	0,021
Ackr3	0,45	0,022
Ctdspl	0,43	0,023
Id1	0,40	0,023
Krt18	0,43	0,023
Gltscr1l	-0,32	0,024
Tgfa	0,36	0,024
Sh3pxd2a	0,37	0,024
Tdh	-0,25	0,024
Lsp1	0,39	0,024
Trim6	-0,24	0,025
Hsp90b1	0,19	0,026
Hmcn2	0,43	0,026
Pml	-0,27	0,026
Fem1b	0,23	0,026
Micall1	0,44	0,028
Esrp2	0,44	0,028
Sox2	-0,27	0,028
Gadd45a	-0,36	0,028
Capg	0,38	0,028
Sema4b	0,29	0,028
Dlx3	0,34	0,029
Mapkbp1	0,44	0,029
Myo1c	0,27	0,029
Gm42927	-0,22	0,029
Vim	-0,26	0,029
Gfpt2	-0,32	0,029
Kctd12	0,40	0,029
Hspa5	0,19	0,030
Rcor2	-0,28	0,030
Tacstd2	0,43	0,031
Hras	0,35	0,032

Gene	log2FC	padj
Tet1	-0,21	0,032
Trp53inp1	-0,27	0,033
Zfp36l2	0,32	0,033
1300017J02R	0,32	0,035
Lrrn4	0,43	0,035
Scn4b	0,33	0,012
Cnn2	0,39	0,013
Vegfa	0,48	0,013
Myo18a	0,37	0,013
Helz2	-0,47	0,014
Tns4	0,42	0,035
Mbnl2	0,36	0,035
Epha1	0,43	0,036
Sema4c	0,39	0,036
Sh3bgrl2	0,37	0,036
Exoc3l2	0,33	0,036
Rev1	-0,33	0,036
Setd7	0,38	0,037
Plek2	0,40	0,037
Trim25	-0,22	0,038
Nrg1	0,43	0,038
Pdpn	0,35	0,038
Hs6st1	0,28	0,040
Tulp4	0,40	0,042
Enc1	-0,23	0,042
Fam89a	0,30	0,042
Dgka	0,35	0,044
Mcm10	-0,27	0,044
S100a11	0,33	0,046
Acp6	-0,34	0,047
Edaradd	0,24	0,048
Rgs14	0,32	0,048
Ifrd1	-0,26	0,048
Adamts1	-0,40	0,049
Calr	0,19	0,0496
Duox1	0,25	0,0496
