# Supplementary material for: p73 is required for vessel integrity controlling endothelial junctional dynamics through Angiomotin
Source: Cell Mol Life Sci. 2022 Oct 1;79(10):535. doi: 10.1007/s00018-022-04560-3 (PMC9525397; doi:10.1007/s00018-022-04560-3)
Supplement: Supplementary file 6 — Supplementary Table 2. List of significant Biological Process (FDR<0.05) in p73KO-iPSCs after TAp73β ectopic expression. (PDF 148 KB) [file 18_2022_4560_MOESM6_ESM.pdf]

Supplementary Table 2. List of significant Biological Process (FDR<0.05) in p73KO-iPSCs after TAp73β ectopic expression. FE: Fold Enrichment. FDR: Fold Discovery Rate

Term	Count	Genes	FE	FDR
GO:0007275~multicellular organism development	115	PDGFA, MMP9, WNT3A, BBX, JAG2, ITPKB, WNT4, GATA6, APOE, ADAM8, EDARADD, DAB2IP, PDPN, PLXNB2, EFNB1, MFGE8, MYH9, MARK4, JUNB, JUP, KRT19, KRT17, TACSTD2, NAV2, VEGFA, DSP, LAMC2, TRP73, DST, SLC38A3, ITGB4, DAG1, CX3CL1, CALR, PXN, SRC, LAMB3, ECE1, P2RY1, IDH1, LAMB1, IVL, COL18A1, MAFB, TGFBR1, IL1RL2, CD276, ITGA3, EPHA1, GAS6, CDKN1C, DLX3, NXN, ITGA6, LAMA5, BMP7, BMP6, HIP1R, DUOX1, SDC4, VGF, MBP, SEMA3F, POU4F3, EEF2K, TGFA, FAM83H, NRG1, HYAL2, SH3PXD2A, SLC25A4, FOXJ1, ACKR3, FLNB, DHRS3, NPTXR, SEMA4C, MDM2, SEMA4B, WNT11, ESRP2, NGFR, WNT9A, ALOX12, PHLDB1, CD109, SFN, FEM1B, CIC, ZFP36L2, COL7A1, ALDH1A3, CYP26B1, BCL9L, CNN2, HS6ST1, HSPA5, NBEAL2, PCSK5, CEBPA, ERF, GJB3, HSPG2, RCAN1, PPP1R13L, RGS14, MICALL1, IL6RA, CAPN1, WNT7B, RDH10, DUSP2, IRF6, ID1, PERP	1,88	1,80E-11
GO:0048731~system development	109	PDGFA, MMP9, WNT3A, BBX, JAG2, ITPKB, WNT4, GATA6, APOE, ADAM8, EDARADD, DAB2IP, PDPN, PLXNB2, EFNB1, MFGE8, MYH9, MARK4, JUNB, JUP, KRT19, KRT17, TACSTD2, NAV2, VEGFA, DSP, LAMC2, TRP73, DST, SLC38A3, ITGB4, DAG1, CX3CL1, CALR, SRC, ECE1, P2RY1, IDH1, LAMB1, IVL, COL18A1, MAFB, IL1RL2, TGFBR1, CD276, ITGA3, EPHA1, GAS6, CDKN1C, DLX3, ITGA6, NXN, LAMA5, BMP7, BMP6, HIP1R, SDC4, VGF, MBP, SEMA3F, POU4F3, EEF2K, TGFA, FAM83H, NRG1, HYAL2, SH3PXD2A, SLC25A4, FOXJ1, ACKR3, FLNB, DHRS3, NPTXR, SEMA4C, MDM2, SEMA4B, WNT11, NGFR, WNT9A, ESRP2, ALOX12, CD109, SFN, FEM1B, CIC, ZFP36L2, ALDH1A3, CYP26B1, BCL9L, CNN2, HS6ST1, HSPA5, NBEAL2, PCSK5, CEBPA, ERF, GJB3, HSPG2, RCAN1, PPP1R13L, RGS14, CAPN1, IL6RA, MICALL1, WNT7B, RDH10, IRF6, ID1, PERP	2,01	1,72E-12

GO:0048869~cellular developmental process	96	HRAS, FERMT3, MMP9, WNT3A, JAG2, ITPKB, SDC4, WNT4, GATA6, APOE, SEMA3F, DMKN, POU4F3, EEF2K, ADAM8, NRG1, EDARADD, HYAL2, DAB2IP, SH3PXD2A, SLC25A4, PDPN, FOXJ1, EFNB1, PLXNB2, KRTDAP, ACKR3, MYH9, FLNB, JUNB, KRT19, NPTXR, KRT17, TACSTD2, KRT14, VEGFA, SEMA4C, MDM2, SEMA4B, DSP, WNT11, TRP73, NGFR, WNT9A, DST, SEPT9, ALOX12, PHLDB1, ITGB4, DAG1, CD109, CX3CL1, SFN, CALR, TAGLN2, FEM1B, PXN, SRC, ZFP36L2, LAMB3, COL7A1, CYP26B1, P2RY1, BCL9L, HS6ST1, HSPA5, LAMB1, NBEAL2, IVL, COL18A1, CEBPA, ERF, MAFB, TGFBR1, IL1RL2, HSPG2, CD276, RCAN1, ITGA3, PPP1R13L, CACNA2D2, EPHA1, RGS14, GAS6, MICALL1, DLX3, CDKN1C, WNT7B, RDH10, NXN, ITGA6, IRF6, ID1, LAMA5, BMP7, BMP6	1,83	1,56E-07
GO:0048513~animal organ development	92	PDGFA, MMP9, WNT3A, BBX, JAG2, ITPKB, SDC4, VGF, MBP, WNT4, GATA6, SEMA3F, POU4F3, TGFA, ADAM8, FAM83H, NRG1, EDARADD, HYAL2, DAB2IP, SH3PXD2A, SLC25A4, PDPN, FOXJ1, EFNB1, PLXNB2, MYH9, FLNB, JUNB, JUP, DHRS3, KRT19, KRT17, TACSTD2, VEGFA, SEMA4C, MDM2, SEMA4B, DSP, WNT11, TRP73, NGFR, ESRP2, WNT9A, ALOX12, SLC38A3, ITGB4, DAG1, CD109, SFN, CALR, FEM1B, CIC, SRC, ZFP36L2, ECE1, ALDH1A3, CYP26B1, IDH1, BCL9L, CNN2, HS6ST1, HSPA5, LAMB1, PCSK5, NBEAL2, IVL, CEBPA, ERF, MAFB, TGFBR1, IL1RL2, GJB3, HSPG2, CD276, ITGA3, RCAN1, PPP1R13L, GAS6, CAPN1, IL6RA, DLX3, CDKN1C, WNT7B, RDH10, ITGA6, IRF6, LAMA5, ID1, PERP, BMP7, BMP6	2,22	4,98E-12
GO:0009653~anatomical structure morphogenesis	85	PDGFA, FERMT3, MMP9, WNT3A, JAG2, SDC4, WNT4, GATA6, APOE, SEMA3F, POU4F3, EEF2K, TGFA, ADAM8, NRG1, EDARADD, HYAL2, DAB2IP, SH3PXD2A, PDPN, FOXJ1, EFNB1, PLXNB2, ACKR3, MFGE8, MYH9, FLNB, JUNB, KRT19, DHRS3, KRT17, TACSTD2, VEGFA, SEMA4C, MDM2, SEMA4B, DSP, WNT11, TRP73, NGFR, ESRP2, WNT9A, DST, SEPT9, ALOX12, PHLDB1, ITGB4, DAG1, CD109, CX3CL1, CALR, FEM1B, SRC, PXN, LAMB3, ECE1, COL7A1, ALDH1A3, CYP26B1, BCL9L, HS6ST1, HSPA5, LAMB1, PCSK5, NBEAL2, COL18A1, ERF, MAFB, TGFBR1, HSPG2, ITGA3, PPP1R13L, EPHA1, CAPN1, DLX3, CDKN1C, RDH10, WNT7B, DUSP2, ITGA6, LAMA5, ID1, PERP, BMP7, BMP6	2,63	3,36E-15

GO:0009888~tissue development	73	PDGFA, WNT3A, MMP9, JAG2, SDC4, WNT4, GATA6, SEMA3F, POU4F3, ADAM8, FAM83H, NRG1, EDARADD, HYAL2, DAB2IP, SLC25A4, PDPN, FOXJ1, EFNB1, PLXNB2, KRTDAP, FLNB, JUNB, KRT17, TACSTD2, VEGFA, KRT14, SEMA4C, MDM2, SEMA4B, DSP, WNT11, TRP73, NGFR, WNT9A, ESRP2, ALOX12, ITGB4, CD109, DAG1, SFN, CALR, TAGLN2, FEM1B, SRC, PXN, LAMB3, COL7A1, ALDH1A3, CYP26B1, BCL9L, LAMB1, IVL, COL18A1, ERF, TGFBR1, HSPG2, CD276, ITGA3, RCAN1, PPP1R13L, GAS6, DLX3, CDKN1C, RDH10, WNT7B, DUSP2, ITGA6, IRF6, LAMA5, ID1, BMP7, BMP6	3,28	1,77E-17
GO:0010646~regulation of cell communication	66	SNCG, HRAS, PDGFA, HIP1R, WNT3A, MMP9, JAG2, ITPKB, VGF, WNT4, GATA6, APOE, TGFA, ADAM8, NRG1, MAPKBP1, VWA2, MVB12B, HYAL2, DAB2IP, SCUBE3, ACKR3, CARD10, DHRS3, VEGFA, SEMA4C, MDM2, WNT11, TRP73, NGFR, WNT9A, EPS8L2, CD109, DAG1, CX3CL1, CALR, FEM1B, SRC, PXN, ECE1, ALDH1A3, CYP26B1, P2RY1, BCL9L, HSPA5, MYO1C, TGFBR1, PTPN13, ITGA3, RCAN1, CACNA2D2, RGS14, GAS6, IL6RA, CDKN1C, HYOU1, WNT7B, ITGA6, NXN, ID1, RGS3, SLC7A1, BMP7, DUSP7, KCTD12, BMP6	2,01	2,14E-05
GO:0009966~regulation of signal transduction	63	HRAS, PDGFA, HIP1R, WNT3A, MMP9, JAG2, ITPKB, WNT4, GATA6, APOE, TGFA, ADAM8, NRG1, MAPKBP1, VWA2, MVB12B, DAB2IP, HYAL2, SCUBE3, ACKR3, CARD10, DHRS3, VEGFA, SEMA4C, MDM2, WNT11, TRP73, NGFR, WNT9A, EPS8L2, CD109, DAG1, CX3CL1, CALR, FEM1B, SRC, PXN, ECE1, ALDH1A3, CYP26B1, P2RY1, BCL9L, HSPA5, MYO1C, TGFBR1, PTPN13, ITGA3, RCAN1, RGS14, GAS6, IL6RA, CDKN1C, HYOU1, WNT7B, ITGA6, NXN, ID1, RGS3, SLC7A1, BMP7, DUSP7, KCTD12, BMP6	2,17	3,14E-06
GO:0006928~movement of cell or subcellular component	62	HRAS, PDGFA, FERMT3, WNT3A, MMP9, FERMT1, AMOTL1, SDC4, CD151, WNT4, APOE, SEMA3F, POU4F3, ADAM8, FAM83H, NRG1, DAB2IP, HYAL2, PDPN, FOXJ1, EFNB1, PLXNB2, CHST3, MYH9, JUP, TACSTD2, VEGFA, CEMIP, SEMA4C, MDM2, SEMA4B, DSP, LAMC2, WNT11, NGFR, DST, MYO18A, ALOX12, SORD, DAG1, ITGB4, CX3CL1, CALR, SRC, PXN, FAT2, P2RY1, CNN2, HSPA5, LAMB1, PLTP, COL18A1, MYO1C, TGFBR1, ITGA3, EPHA1, GAS6, WNT7B, ITGA6, LAMA5, SCN4B, BMP7	3,05	1,89E-12

GO:0048870~cell motility	52	HRAS, PDGFA, MMP9, FERMT3, FERMT1, SDC4, CD151, AMOTL1, WNT4, APOE, SEMA3F, ADAM8, NRG1, FAM83H, DAB2IP, HYAL2, FOXJ1, PDPN, PLXNB2, EFNB1, MYH9, JUP, TACSTD2, VEGFA, CEMIP, SEMA4C, SEMA4B, MDM2, LAMC2, WNT11, MYO18A, ALOX12, SORD, DAG1, ITGB4, CX3CL1, CALR, SRC, P2RY1, FAT2, CNN2, HSPA5, LAMB1, PLTP, COL18A1, MYO1C, TGFBR1, ITGA3, EPHA1, GAS6, ITGA6, LAMA5	3,28	5,74E-11
GO:0016477~cell migration	50	HRAS, PDGFA, MMP9, FERMT3, FERMT1, SDC4, CD151, AMOTL1, WNT4, APOE, SEMA3F, NRG1, FAM83H, ADAM8, DAB2IP, HYAL2, FOXJ1, PDPN, PLXNB2, EFNB1, MYH9, JUP, TACSTD2, VEGFA, SEMA4C, CEMIP, SEMA4B, MDM2, LAMC2, WNT11, MYO18A, ALOX12, DAG1, ITGB4, CX3CL1, CALR, SRC, P2RY1, FAT2, CNN2, HSPA5, LAMB1, COL18A1, MYO1C, TGFBR1, ITGA3, EPHA1, GAS6, ITGA6, LAMA5	3,54	1,08E-11
GO:2000026~regulation of multicellular organismal development	50	PDGFA, MMP9, WNT3A, ITPKB, WNT4, GATA6, APOE, SEMA3F, EEF2K, NRG1, ADAM8, DAB2IP, SLC25A4, FOXJ1, PLXNB2, KRT17, TACSTD2, VEGFA, SEMA4C, SEMA4B, MDM2, WNT11, WNT9A, NGFR, TRP73, ALOX12, PHLDB1, DAG1, CD109, SFN, CX3CL1, CALR, ZFP36L2, CYP26B1, BCL9L, HSPA5, MAFB, IL1RL2, TGFBR1, CD276, ITGA3, EPHA1, GAS6, RGS14, CDKN1C, WNT7B, LAMA5, ID1, BMP7, BMP6	2,25	9,77E-05
GO:0050790~regulation of catalytic activity	48	FXYD3, HRAS, PDGFA, HIP1R, WNT3A, MMP9, CD109, DAG1, CX3CL1, SFN, FEM1B, SDC4, 1300017J02RIK, SRC, PXN, WNT4, COL7A1, APOE, TGFA, HSPA5, ADAM8, NRG1, MAPKBP1, DAB2IP, HYAL2, FOXJ1, PLXNB2, TGFBR1, RCAN1, EPHA1, RGS14, GAS6, CARD10, WNT7B, HSP90B1, ITGA6, VEGFA, CEMIP, SETD7, MDM2, WNT11, TRP73, NGFR, WNT9A, BMP7, PERP, DUSP7, ALOX12	2,45	1,41E-05
GO:0098609~cell-cell adhesion	46	LIMA1, ASS1, FERMT3, WNT3A, JAG2, PDIA6, ITPKB, CX3CL1, SFN, TAGLN2, SDC4, CD151, SRC, PAK6, ZFP36L2, WNT4, FAT2, CYP26B1, IDH1, CNN2, FAM129B, ADAM8, LAMB1, ERF, FOXJ1, PDPN, MAFB, EFNB1, PLXNB2, IL1RL2, CD276, CSRP1, MYH9, FLNB, PPP1R13L, JUP, WNT7B, ITGA6, TACSTD2, PKP3, CAPG, VEGFA, DSP, BMP7, PERP, ALOX12	3,55	1,74E-10
GO:0051270~regulation of cellular component movement	44	HRAS, PDGFA, FERMT3, MMP9, WNT3A, DAG1, CX3CL1, CALR, AMOTL1, SDC4, SRC, WNT4, APOE, SEMA3F, CNN2, HSPA5, ADAM8, FAM83H, NRG1, LAMB1, COL18A1, DAB2IP, HYAL2, MYO1C, PDPN, PLXNB2, TGFBR1, CHST3, ITGA3, EPHA1, GAS6, JUP, ITGA6, TACSTD2, LAMA5, VEGFA, CEMIP, SEMA4C, DSP, MDM2, SEMA4B, LAMC2, WNT11, ALOX12	4,42	3,44E-13

GO:0030030~cell projection organization	41	LIMA1, HRAS, PDGFA, WNT3A, DAG1, ITGB4, SDC4, SRC, PXN, APOE, SEMA3F, POU4F3, EEF2K, HSPA5, NRG1, LAMB1, DAB2IP, FOXJ1, EFNB1, PLXNB2, TGFBR1, ITGA3, MYH9, MARK4, MICALL1, WNT7B, NPTXR, ITGA6, TACSTD2, ID1, LAMA5, VEGFA, CAPG, SEMA4C, MDM2, SEMA4B, NGFR, BMP7, DST, EPS8L2, SEPT9	2,50	1,50E-04
GO:2000145~regulation of cell motility	40	HRAS, PDGFA, FERMT3, MMP9, DAG1, CX3CL1, CALR, AMOTL1, SDC4, SRC, WNT4, APOE, SEMA3F, CNN2, HSPA5, ADAM8, FAM83H, NRG1, LAMB1, COL18A1, DAB2IP, HYAL2, MYO1C, PDPN, PLXNB2, TGFBR1, ITGA3, EPHA1, GAS6, ITGA6, LAMA5, TACSTD2, VEGFA, CEMIP, SEMA4C, MDM2, SEMA4B, LAMC2, WNT11, ALOX12	4,34	1,96E-11
GO:0098602~single organism cell adhesion	37	ASS1, FERMT3, WNT3A, JAG2, PDIA6, ITPKB, CALR, SDC4, CD151, PXN, SRC, ZFP36L2, WNT4, CYP26B1, ADAM8, LAMB1, ERF, FOXJ1, PDPN, MAFB, EFNB1, IL1RL2, CD276, CSRP1, MYH9, EPHA1, JUP, WNT7B, ITGA6, LAMA5, TACSTD2, PKP3, VEGFA, DSP, BMP7, PERP, ALOX12	3,95	4,19E-09
GO:0009968~negative regulation of signal transduction	33	MMP9, WNT3A, DAG1, CD109, CX3CL1, CALR, SRC, WNT4, APOE, CYP26B1, BCL9L, HSPA5, DAB2IP, HYAL2, TGFBR1, ACKR3, ITGA3, RGS14, GAS6, HYOU1, DHRS3, WNT7B, NXN, ITGA6, RGS3, VEGFA, MDM2, WNT11, TRP73, NGFR, WNT9A, BMP7, DUSP7	2,73	5,82E-04
GO:0010648~negative regulation of cell communication	33	MMP9, WNT3A, DAG1, CD109, CX3CL1, CALR, SRC, WNT4, APOE, CYP26B1, BCL9L, HSPA5, DAB2IP, HYAL2, TGFBR1, ACKR3, ITGA3, RGS14, GAS6, HYOU1, DHRS3, WNT7B, NXN, ITGA6, RGS3, VEGFA, MDM2, WNT11, TRP73, NGFR, WNT9A, BMP7, DUSP7	2,42	8,22E-03
GO:0023057~negative regulation of signaling	33	MMP9, WNT3A, DAG1, CD109, CX3CL1, CALR, SRC, WNT4, APOE, CYP26B1, BCL9L, HSPA5, DAB2IP, HYAL2, TGFBR1, ACKR3, ITGA3, RGS14, GAS6, HYOU1, DHRS3, WNT7B, NXN, ITGA6, RGS3, VEGFA, MDM2, WNT11, TRP73, NGFR, WNT9A, BMP7, DUSP7	2,41	8,82E-03
GO:0016337~single organismal cell-cell adhesion	32	ASS1, FERMT3, WNT3A, JAG2, PDIA6, ITPKB, SDC4, CD151, SRC, ZFP36L2, WNT4, CYP26B1, ADAM8, LAMB1, ERF, FOXJ1, PDPN, MAFB, EFNB1, IL1RL2, CD276, CSRP1, MYH9, JUP, WNT7B, ITGA6, PKP3, VEGFA, DSP, PERP, BMP7, ALOX12	3,68	1,02E-06
GO:0051272~positive regulation of cellular component movement	31	HRAS, PDGFA, FERMT3, MMP9, CX3CL1, CALR, AMOTL1, SRC, SEMA3F, HSPA5, ADAM8, FAM83H, LAMB1, COL18A1, DAB2IP, MYO1C, PDPN, TGFBR1, CHST3, ITGA3, EPHA1, GAS6, ITGA6, VEGFA, CEMIP, SEMA4C, SEMA4B, MDM2, LAMC2, WNT11, ALOX12	5,58	6,51E-11

GO:2000147~positive regulation of cell motility	30	HRAS, PDGFA, FERMT3, MMP9, CX3CL1, CALR, AMOTL1, SRC, SEMA3F, HSPA5, ADAM8, FAM83H, LAMB1, COL18A1, DAB2IP, MYO1C, PDPN, TGFBR1, ITGA3, EPHA1, GAS6, ITGA6, VEGFA, CEMIP, SEMA4C, SEMA4B, MDM2, LAMC2, WNT11, ALOX12	5,53	2,34E-10
GO:0040017~positive regulation of locomotion	30	HRAS, PDGFA, FERMT3, MMP9, CX3CL1, CALR, AMOTL1, SRC, SEMA3F, HSPA5, ADAM8, FAM83H, LAMB1, COL18A1, DAB2IP, MYO1C, PDPN, TGFBR1, ITGA3, EPHA1, GAS6, ITGA6, VEGFA, CEMIP, SEMA4C, SEMA4B, MDM2, LAMC2, WNT11, ALOX12	5,37	4,71E-10
GO:0030029~actin filament-based process	28	HRAS, LIMA1, PDGFA, HIP1R, CALR, SDC4, SRC, WNT4, CNN2, MYO1C, FOXJ1, TGFBR1, CORO6, CORO7, CSRP1, MYH9, EPHA1, FLNB, JUP, HSP90B1, KRT19, TACSTD2, ID1, CAPG, SCN4B, DSP, WNT11, MYO18A	3,62	2,21E-05
GO:0001568~blood vessel development	27	PDGFA, MMP9, CX3CL1, WNT4, GATA6, APOE, TGFA, HS6ST1, ADAM8, PCSK5, COL18A1, DAB2IP, TGFBR1, HSPG2, ACKR3, MFGE8, MYH9, EPHA1, JUNB, DLX3, WNT7B, VEGFA, MDM2, WNT11, NGFR, BMP7, ALOX12	3,58	5,62E-05
GO:0048514~blood vessel morphogenesis	23	COL18A1, DAB2IP, PDGFA, TGFBR1, MMP9, HSPG2, ACKR3, MFGE8, CX3CL1, MYH9, EPHA1, JUNB, WNT4, WNT7B, GATA6, APOE, VEGFA, TGFA, WNT11, HS6ST1, NGFR, ADAM8, ALOX12	3,62	6,32E-04
GO:0031589~cell-substrate adhesion	21	FERMT3, ITGB4, DAG1, BCAM, ITGA3, NID2, CALR, SDC4, EPHA1, SRC, PXN, GAS6, WNT4, ITGA6, TACSTD2, LAMA5, ID1, VEGFA, ADAM8, LAMB1, VWA2	6,00	5,32E-07
GO:0001525~angiogenesis	19	COL18A1, DAB2IP, PDGFA, TGFBR1, MMP9, HSPG2, ACKR3, MFGE8, CX3CL1, MYH9, EPHA1, WNT7B, GATA6, VEGFA, TGFA, HS6ST1, NGFR, ADAM8, ALOX12	3,63	8,08E-03
GO:0045785~positive regulation of cell adhesion	18	WNT3A, IL1RL2, EFNB1, DAG1, CD276, ITPKB, ITGA3, CX3CL1, CALR, SDC4, EPHA1, SRC, WNT4, ITGA6, VEGFA, ADAM8, BMP7, ALOX12	4,18	2,43E-03
GO:0022407~regulation of cell-cell adhesion	17	ASS1, FOXJ1, FERMT3, WNT3A, EFNB1, IL1RL2, CD276, ITPKB, SDC4, SRC, WNT4, ITGA6, VEGFA, CYP26B1, ADAM8, BMP7, ALOX12	3,80	1,72E-02
GO:0032970~regulation of actin filament-based process	16	LIMA1, HRAS, MYO1C, HIP1R, TGFBR1, MYH9, SDC4, EPHA1, JUP, WNT4, TACSTD2, ID1, CAPG, DSP, WNT11, CNN2	3,94	2,23E-02
GO:0034330~cell junction organization	14	MYO1C, TGFBR1, ITGB4, PTPN13, SDC4, SRC, PXN, JUP, WNT4, PKP3, VEGFA, DSP, WNT11, PERP	5,97	1,03E-03
GO:0043062~extracellular structure organization	14	COL18A1, PHLDB1, MMP9, WNT3A, TGFBR1, DAG1, HSPG2, NID2, GAS6, LAMB3, LAMA5, LAMC2, VWA1, LAMB1	5,19	4,95E-03
